# P25 and P37 proteins encoded by firespike leafroll-associated virus are viral suppressors of RNA silencing

**DOI:** 10.3389/fmicb.2022.964156

**Published:** 2022-08-16

**Authors:** Yaqin Wang, Hui Liu, Zhanqi Wang, Yushuang Guo, Tao Hu, Xueping Zhou

**Affiliations:** ^1^State Key Laboratory of Rice Biology, Institute of Biotechnology, Zhejiang University, Hangzhou, China; ^2^Key Laboratory of Vector Biology and Pathogen Control of Zhejiang Province, College of Life Sciences, Huzhou University, Huzhou, China; ^3^Key Laboratory of Molecular Genetics, Guizhou Academy of Tobacco Science, Guiyang, China; ^4^State Key Laboratory for Biology of Plant Diseases and Insect Pests, Institute of Plant Protection, Chinese Academy of Agricultural Sciences, Beijing, China

**Keywords:** ampelovirus, firespike leafroll-associated virus, suppressor, RNA silencing, PTGS, TGS

## Abstract

Firespike leafroll-associated virus (FLRaV) is a major pathogen associated with firespike (*Odontonema tubaeforme*) leafroll disease. Phylogenetic analysis showed that FLRaV possesses typical traits of subgroup II members of ampeloviruses, but encodes two additional proteins, P25 and P37. Here, we determined the microfilament localization of P25 protein. Posttranscriptional gene silencing (PTGS) assay showed that both FLRaV P25 and P37 were able to suppress the local and systemic PTGS and FLRaV P25 was capable of suppressing the green fluorescent protein (GFP) gene silencing triggered by both sense RNA-induced PTGS (S-PTGS) and inverted repeat RNA-induced PTGS (IR-PTGS). In contrast, FLRaV P37 was only able to inhibit the GFP silencing triggered by the S-PTGS but not the IR-PTGS. In the transcriptional gene silencing (TGS) assay, only FLRaV P25 was found to be able to reverse established TGS-mediated silencing of GFP in 16-TGS plants. We also found that FLRaV P25 could aggravate the disease symptom and viral titer of potato virus X in *N. benthamiana*. These results suggest that FLRaV P25 and P37 may have crucial roles in overcoming host RNA silencing, which provides key insights into our understanding of the molecular mechanisms underlying FLRaV infection.

## Introduction

During plant-virus interactions, viral double-stranded RNAs (dsRNAs) are recognized and cleaved by the host Dicer-like proteins into 21–24 nt virus-derived small-interfering RNAs (vsiRNAs) (Guo et al., 2019; Jin et al., 2021). These vsiRNAs subsequently interact with argonaute proteins and form the RNA-induced silencing complexes to target the viral mRNAs and/or genomes for posttranscriptional gene silencing (PTGS) or transcriptional gene silencing (TGS) (Yang and Li, 2018; Jin et al., 2021). Numerous studies have documented that plant RNA silencing is an essential immune system to defend against viral infections (Szittya and Burgyán, 2013; Guo et al., 2019; Jin et al., 2021, 2022). Frequently, plants defective in RNA silencing become susceptible to viral infections (Carbonell et al., 2012; Qin et al., 2017; Kørner et al., 2018; Wang et al., 2021). To withstand the host RNA silencing, plant viruses have evolved various viral suppressors of RNA silencing (VSRs) to impede this response at different steps (Yang and Li, 2018; Hussain et al., 2021; Jin et al., 2021, 2022), such as P19 of tombusviruses (Chapman et al., 2004), 2b of cucumoviruses (Zhang et al., 2006), βC1, V2, and C4 proteins of geminiviruses (Cui et al., 2005; Glick et al., 2008; Ismayil et al., 2018), VPg and HC-Pro proteins of potyviruses (Rajamäki et al., 2014; Hafrén et al., 2018), and P3 of tenuiviruses (Chen et al., 2020; Jiang et al., 2021).

Ampeloviruses (family *Closteroviridae*) are plant viruses with positive-single stranded RNA genomes, causing devastating diseases in fruit crops and stock nurseries around the globe (Fuchs et al., 2020). The genome length varies among the members of the genus *Ampelovirus*, while their genomic organization shares conserved genetic modules. The open reading frame (ORF) 1a and b of ampelovirus that encode two replication-associated polyproteins constitute the replication gene block (RGB) (Ito and Nakaune, 2016). The quintuple gene block (QGB) consists of five ORFs that sequentially encode a polypeptide with a conserved hydrophobic region, two putative proteins that contain a heat shock protein 70 (HSP70) domain and a HSP90 domain, respectively, a coat protein, and a minor coat protein (Rubio et al., 2013; Ito and Nakaune, 2016; Dong et al., 2022). This genus can be further divided into two subgroups based on genome size, gene structure, and sequence similarity. Subgroup I viruses have larger genomes and additional ORFs coding for accessory proteins. In contrast, subgroup II viruses have smaller genomes and lack a variable coding region at 3’-end of the genome.

Firespike leafroll-associated virus (FLRaV) is a newly identified *Ampelovirus* from firespike (*Odontonema tubaeforme*) with curly leaf and chlorosis symptoms (Wang et al., 2020). Phylogenetic analysis showed that FLRaV possesses typical traits of *Ampelovirus* subgroup II, such as reduced genome size and a small intergenic region between two gene modules. However, unlike other members in this subgroup, the FLRaV virus genome encodes two additional proteins downstream of the QGB, a putative 25.0-kDa protein (P25) with a nucleic acid-binding domain and two clathrin-binding motifs, and a putative 37.0-kDa protein (P37) with a high similarity to the MYB transcription factors (Wang et al., 2020).

To further explore the biological functions of P25 and P37 proteins, in this study, we showed that FLRaV P25 is localized in the microfilament of the cytoskeleton and nucleus, while FLRaV P37 is discontinuously distributed in the cytosol and nucleus. Both P25 and P37 are capable of suppressing both local and systemic PTGS of green fluorescent protein (GFP) gene mainly in a sense RNA-dependent manner. Furthermore, FLRaV P25 can also suppress the methylation-mediated TGS of GFP in 16-TGS plants. Transient expression of FLRaV P25 in *Nicotiana benthamiana* can aggravate the disease symptom and viral titer of potato virus X (PVX). Our results present the first evidence that the P25 and P37 of FLRaV are VSRs and they are capable of suppressing the host RNA silencing at both PTGS and TGS levels.

## Materials and methods

### Plant materials

Wild-type *N. benthamiana* plants, transgenic *N. benthamiana* lines 16c (Ruiz et al., 1998; Li et al., 2014) and 16-TGS (Buchmann et al., 2009) were grown in a greenhouse at 25°C with a 16/8-h (light/dark) photoperiod as described previously (Zhong et al., 2017a). Seedlings at the 4- to 6-leaf stage were utilized for the experiments as described by Yang et al. (2011).

### Subcellular localization analysis of FLRaV P25 and P37

To construct the plasmids for subcellular localization, ORFs of FLRaV P25 and P37 genes were cloned by fusing them to the N-terminus of GFP under a CaMV 35S (35S) promoter to produce 35S:P25-GFP and 35S:P37-GFP, respectively, as described previously (Zhong et al., 2017a). A plasmid expressing the actin-binding domain 2 of *Arabidopsis* fimbrin 1 with mCherry (ABD2-mCherry) was constructed as described previously (Feng et al., 2013; Han et al., 2013). The primers used for plasmid construction are listed in Supplementary Table 1. *Agrobacterium*-mediated infiltration of *N. benthamiana* leaves was carried out as described previously (Yang et al., 2011; Zhong et al., 2017a). At 48 h post infiltration (hpi), the agroinfiltrated leaves of *N. benthamiana* were examined *via* laser scanning confocal microscope (Olympus, Tokyo, Japan). The latrunculin A (Lat-A) (Merck Sigma-Aldrich, Milwaukee, WI, United States) treatment was performed as described previously (Morton et al., 2000; Feng et al., 2013). Equivalent dilution of dimethylsulfoxide (DMSO) (Merck Sigma-Aldrich, Milwaukee, WI, United States) was used as the control.

### PTGS suppression analysis of FLRaV P25 and P37

To transiently express FLRaV P25 and P37 genes, the ORFs were cloned into the *Xho*I-*Bam*HI sites of the binary vector pGD (Goodin et al., 2002) to generate pGD:P25 and pGD:P37, respectively. All the produced recombinant pGD vectors were electroporated into the *Agrobacterium tumefaciens* strain EHA105 as described previously (Zhong et al., 2017b). For local and systemic PTGS experiments, classic two-component PTGS assays were carried out by co-infiltration of 35S:GFP with the pGD:P25 or pGD:P37 into leaves of *N. benthamiana* 16c seedlings, as described previously (Li et al., 2014). *A. tumefaciens* harboring an empty pGD (pGD:Vec) or P19 of tomato bushy stunt virus (TBSV P19) (Qu and Morris, 2002) served as the negative and positive controls, respectively. Sense RNA-induced PTGS (S-PTGS) and inverted repeat RNA-induced PTGS (IR-PTGS) assays using a three-component approach were carried out according to the methods established by Li et al. (2014, 2015).

### TGS suppression analysis of FLRaV P25 and P37

To produce the PVX expression constructs, the ORFs of FLRaV P25 and P37 genes fused with a 3 × HA tag were cloned into the *Asc*I-*Sal*I sites of the PVX-based expression vector pGR106 (Lu et al., 2003). All the constructed PVX vectors were electroporated into the *A. tumefaciens* strain GV3101 as described previously (Hu et al., 2020). For TGS experiments, *A. tumefaciens* cultures containing the constructed PVX vectors were infiltrated into leaves of *N. benthamiana* 16-TGS seedlings, as described previously (Yang et al., 2011; Zhong et al., 2017a; Zhao et al., 2022a).

### RNA extraction and quantitative real time PCR analysis

Total RNA extraction from infiltrated *N. benthamiana* leaves was carried out using a TRIzol reagent (Invitrogen, Carlsbad, CA, United States) as described previously (Zhao et al., 2022a). cDNA was reverse transcribed from 1°μg of total RNA using a PrimeScript™ RT Master Mix (TaKaRa, Shiga, Japan) as described previously (Zhong et al., 2017a). qRT-PCR was performed on a LightCycler^^®^^ 480 II instrument (Roche Diagnostics, Mannheim, Germany) as described previously (Zhong et al., 2017b). For each candidate gene, the relative expression levels were calculated using a 2^–ΔΔ*Ct*^ method (Livak and Schmittgen, 2001) and the results were averages of three biological replicates. *N. benthamiana actin 2* (*NbACT2*) was utilized as an internal control as described by Zhong et al. (2017a) and Gui et al. (2022). The primers used for qRT-PCR analysis are listed in Supplementary Table 1.

### Protein extraction and western blot analysis

Leaf samples (approximately 100 mg) were ground into powders with N2 and homogenized in 300°μL extraction buffer containing 50 mM Tris–HCl (pH 6.8), 9°M urea, 4.5% SDS, and 7.5%-mercaptoethanol (Merck Sigma-Aldrich, Milwaukee, WI, United States) (Xiong et al., 2008; Zhao et al., 2022a). The resulting supernatants were subjected to WB analysis. Immunoblotting was carried out as described previously (Li et al., 2014; Zhong et al., 2017a). The monoclonal antibodies to GFP, HA, and Actin were purchased from Abmart (Shanghai, China), and the monoclonal antibody against PVX CP was produced in-house. Chemiluminescent signals were detected using a Luminata™ Forte Western HRP Substrate (Merck Millipore, Watford, United Kingdom) according to the manufacturer’s instructions.

### Genomic DNA extraction and chop-PCR analysis

Total genomic DNA was isolated from leaf samples using a Super Plant Genomic DNA Kit (Tiangen, Beijing, China) according to the manufacturer’s instructions. Approximately 300°ng genomic DNA was digested with a methylation-sensitive restriction enzyme *Hpa*II, as described previously (Li et al., 2020). Chop-PCR was carried out as described previously (Zhong et al., 2017a; Kong et al., 2020).

### Statistical analysis

The data are given as means ± standard deviation (SD) of three independent biological replicates. For significance analysis, a Tukey’s test was performed for comparison of the individual means using the SPSS software (v.24.0, SPSS Inc., Chicago, IL, United States) and values were considered significantly different at a *P*-value of < 0.05.

## Results

### Subcellular localization of P25 and P37 proteins of FLRaV

**FIGURE 1:**
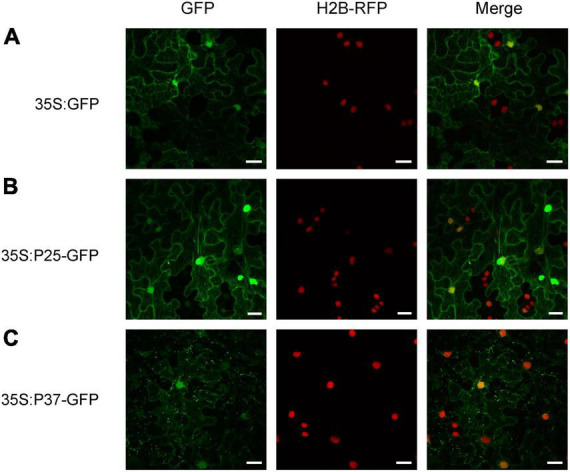
Sublocalization of the P25 and P37 proteins of firespike leafroll-associated virus (FLRaV). **(A)** Localization of green fluorescent protein (GFP) in H2B-RFP *Nicotiana benthamiana* leaf epidermal cells at 48 h post infiltration (hpi). **(B)** Localization of FLRaV P25-GFP fusion protein in H2B-RFP *N. benthamiana* leaf epidermal cells at 48 hpi. **(C)** Localization of FLRaV P37-GFP fusion protein in H2B-RFP *N. benthamiana* leaf epidermal cells at 48 hpi. Histone 2B-RFP (H2B-RFP) was served as a marker for the nucleus. Bar = 20 μm. Images are reconstructed by superposition of a series of confocal optical sections.

**FIGURE 2:**
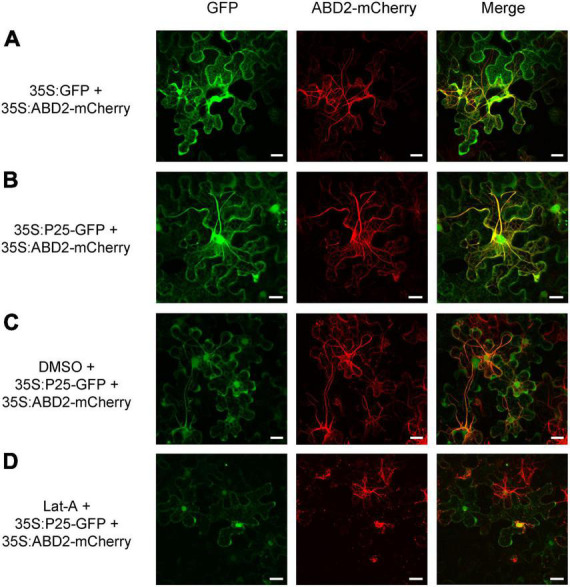
Colocalization of firespike leafroll-associated virus (FLRaV) P25 and P37 proteins with the actin-binding domain 2 of *Arabidopsis* fimbrin 1 (ABD2). **(A)** Colocalization of the green fluorescent protein (GFP) and actin-binding domain 2 of Arabidopsis fimbrin 1 with mCherry (ABD2-mCherry) in wild-type *Nicotiana benthamiana* leaf epidermal cells at 48 h post infiltration (hpi). **(B)** Colocalization of the FLRaV P25-GFP and ABD2-mCherry in wild-type *N. benthamiana* leaf epidermal cells at 48 hpi. **(C,D)** Colocalization of FLRaV P25-GFP with ABD2-mCherry in wild-type *N. benthamiana* leaf epidermal cells at 48 hpi under dimethylsulfoxide (DMSO) or 10 μM latrunculin A (Lat-A) treatments, respectively. Bar = 20 μm. Images are reconstructed by superposition of a series of confocal optical sections.

To obtain further insight into the function of FLRaV P25 and P37 during viral infection, full-length open reading frames encoding P25 and P37 were fused to the green fluorescent protein (GFP), respectively, and expressed in transgenic *N. benthamiana* plants harboring red fluorescent protein (RFP)-H2B *via* agroinfiltration. As shown in Figure 1A, *N. benthamiana* epidermal cells transiently expressing GFP only (35S:GFP) exhibited dispersed fluorescence. In contrast, radial or filamentous GFP fluorescence was observed in both cytosol and nucleus in *N. benthamiana* epidermal cells expressing 35S:P25-GFP, and the P37-GFP fluorescence was detected in both nuclei and cytoplasm, with granules spread in the cytoplasm (Figures 1B,C). Since FLRaV P25 protein had two putative clathrin-binding motifs and was localized to the filamentous structures in the cytosol, we next examined whether P25 was associated with a cytoskeleton (Dell’Angelica, 2001). To test this, the marker protein ABD2 was used to designate the microfilament (Dyachok et al., 2014). As expected, compared with the control (Figure 2A), *N. benthamiana* epidermal cells co-expressing FLRaV P25-GFP and ABD2-mCherry showed yellow fluorescence derived from a superposition of green and red fluorescence (Figure 2B). To further confirm the co-localization of P25-GFP and ABD2-mCherry, the *N. benthamiana* leaves were treated with Lat-A to prevent microfilament polymerization as described previously (Morton et al., 2000). Compared with the *N. benthamiana* leaves treated with DMSO (Figure 2C), Lat-A treatment inhibited the radial fluorescence of both FLRaV P25-GFP and ABD2-mCherry (Figure 2D). Collectively, these results suggest that FLRaV P25 and P37 have different subcellular localization patterns and FLRaV P25 is able to localize in the microfilament of the cytoskeleton.

### FLRaV P25 and P37 function as suppressors of PTGS of *GFP*

**FIGURE 3:**
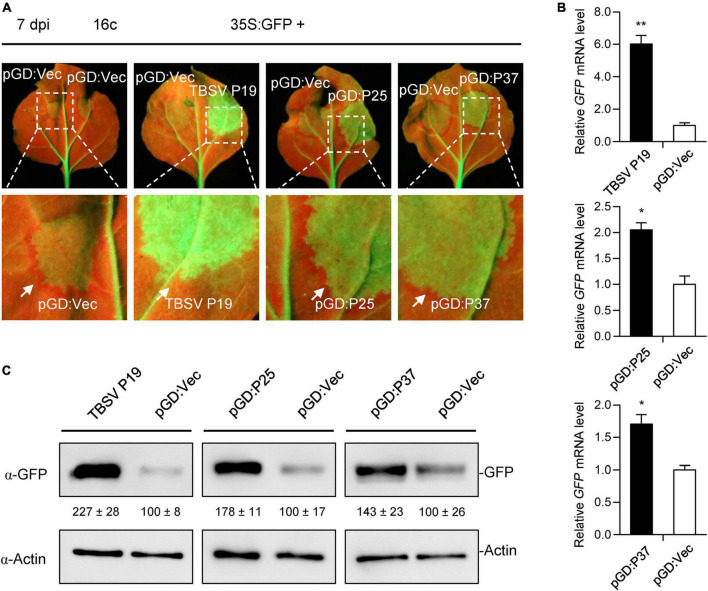
Firespike leafroll-associated virus (FLRaV) P25 and P37 can inhibit the post-transcriptional gene silencing (PTGS) of local green fluorescent protein (GFP) in 16c *Nicotiana benthamiana*. **(A)** Leaves of 16c *N. benthamiana* seedlings were co-infiltrated with *Agrobacterium tumefaciens* harboring the binary vector expressing GFP (35S:GFP) and either an empty pGD vector (pGD:Vec), TBSV P19, pGD:P25, or pGD:P37 at 7 days post infiltration (dpi). Leaves of 16c *N. benthamiana* seedlings co-infiltrated with A. tumefaciens harboring the 35S:GFP plus pGD:Vec or TBSV P19 were served as negative and positive controls, respectively. White arrows indicate the red ring around the infiltrated patches. **(B)** Quantitative real time PCR (qRT-PCR) analysis of the accumulation of GFP mRNA in leaf patches shown in Figure 3A. *NbACT2* was utilized as the internal control. Data are presented as means ± SD of three biological replicates. Means with asterisks are significantly different (Student’s *t*-test; *P* < 0.05). **(C)** Western blot (WB) analysis of the accumulation of GFP protein in leaf patches shown in Figure 3A. GFP protein was detected using an antibody against GFP (α-GFP). WB analysis of actin with an α-Actin was served as the loading control.

Compared with proteins encoded by similarly located ORFs of other members of the family *Closteroviridae*, FLRaV P25 and P37 might be involved in the suppression of host RNA interference (Gouveia et al., 2012; Li et al., 2018a). To test this, we first investigated their ability to inhibit the local GFP silencing using transgenic *N. benthamiana* 16c lines (Ruiz et al., 1998; Li et al., 2014). For this purpose, the ORFs of P25 and P37 genes of FLRaV were amplified and cloned into a pGD binary vector (Goodin et al., 2002) to produce pGD:P25 and pGD:P37 constructs, respectively. The local PTGS assay was carried out as described previously (Li et al., 2014; Zhong et al., 2017a; Zhao et al., 2022a). 16c seedlings co-infiltrated with *A. tumefaciens* harboring 35S:GFP and *A. tumefaciens* containing pGD:Vec or TBSV P19 (Qu and Morris, 2002) were served as the negative and positive controls, respectively. As anticipated, leaves of 16c seedlings co-agroinfiltrated with 35S:GFP and TBSV P19 showed extremely strong GFP fluorescence under UV-B light due to inhibition of GFP RNA silencing by P19 at the 7th day after inoculation (Figure 3A). Similar to the positive control, leaves of 16c seedlings co-agroinfiltrated with 35S:GFP plus pGD:P25 or pGD:P37 elicited relatively strong GFP fluorescence under UV-B light at 7-day post inoculation (Figure 3A). These observations indicated that FLRaV P25 and P37 were able to suppress the PTGS of GFP. Consistently, qRT-PCR and WB analyses confirmed that higher fluorescence detected in leaves of 16c seedlings co-infiltrated with the pGD:P25 or pGD:P37 together with 35S:GFP resulted from an increase in GFP mRNA and protein accumulation (Figures 3B,C). These results indicate that FLRaV P25 and P37 can suppress the local GFP silencing in 16c *N. benthamiana*. The silencing signal from the agroinfiltrated area is able to move to 10 to 15 neighboring cells, which causes the reduction of GFP expression in these cells, and can be visualized as a red ring around the infiltrated region under UV-B light (Himber et al., 2003). In agreement with previous research, TBSV P19 effectively inhibited short range movement of silencing signal (Himber et al., 2003), while clear red rings were developed around the regions infiltrated with 35S:GFP plus pGD:P25, pGD:P37 or the negative control, suggesting that neither P25 nor P37 could suppress the cell-to-cell spread of RNA silencing (Figure 3A). These results indicate that the suppression of PTGS for P25 and P37 are not through direct binding of siRNAs.

**FIGURE 4:**
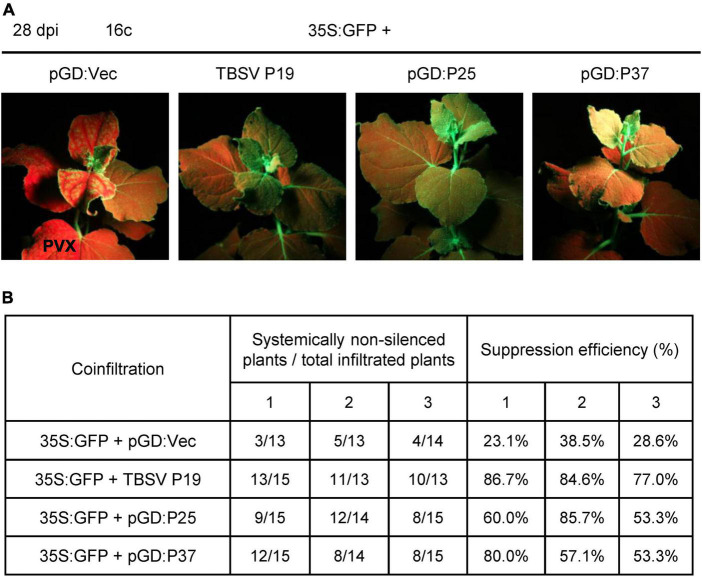
Firespike leafroll-associated virus (FLRaV) P25 and P37 can inhibit the post-transcriptional gene silencing (PTGS) of systemic green fluorescent protein (GFP) in 16c *Nicotiana benthamiana*. **(A)** 16c *N. benthamiana* seedlings were co-infiltrated with *Agrobacterium tumefaciens* containing the binary vector expressing GFP (35S:GFP) plus pGD:Vec, TBSV P19, pGD:P25, or pGD:P37 at 28 days post infiltration (dpi). Leaves of 16c *N. benthamiana* seedlings co-infiltrated with *A. tumefaciens* containing the 35S:GFP plus pGD:Vec or TBSV P19 were served as negative and positive controls, respectively. **(B)** Statistics of 16c *N. benthamiana* seedlings co-agroinfiltrated with the 35S:GFP plus pGD:Vec, TBSV P19, pGD:P25, or pGD:P37 showed systemic GFP RNA silencing at 28 dpi. At least 13 *N. benthamiana* seedlings were utilized in each independent systemic silencing experiment.

Furthermore, we also examined the ability of FLRaV P25 and P37 to impede the systemic silencing established by GFP in 16c plants. To achieve this goal, we co-expressed 35S:GFP together with FLRaV P25 or P37 in 16c *N. benthamiana* seedlings *via* agroinfiltration as described previously (Li and Wang, 2022; Zhao et al., 2022a). At 28 dpi, the agroinfiltrated seedlings were photographed under UV-B light. Compared with the vector control (pGD:Vec), 16c seedlings agroinfiltrated with 35S:GFP plus pGD:P25 or pGD:P37 displayed characteristic GFP fluorescence in the young systemic leaves under UV-B light, which were similar to these seedlings co-infiltrated with 35S:GFP and TBSV P19 (Figure 4A). These results indicated that both FLRaV P25 and P37 can suppress GFP-established systemic RNA silencing in the systemic leaves. Additionally, the statistical analysis indicated that systemic silencing of GFP occurred in 23.1–38.5% of the seedlings agroinfiltrated with 35S:GFP plus pGD:Vec at 28 dpi (Figure 4B). In contrast, 53.3–85.7% and 53.3–80.0% of the 16c seedlings agroinfiltrated with 35S:GFP plus pGD:P25 or pGD:P37 were able to suppress the spread of *GFP* gene silencing in the systemic leaves, which were slightly lower than that in these seedlings agroinfiltrated with 35S:GFP plus TBSV P19 (77.0–86.7%) (Figure 4B). These results suggest that FLRaV P25 and P37 are suppressors of PTGS and are able to restrain *GFP* gene silencing at both local and systemic levels in 16c *N. benthamiana*.

### FLRaV P25 and P37 suppress the PTGS mainly in a sense RNA manner

**FIGURE 5:**
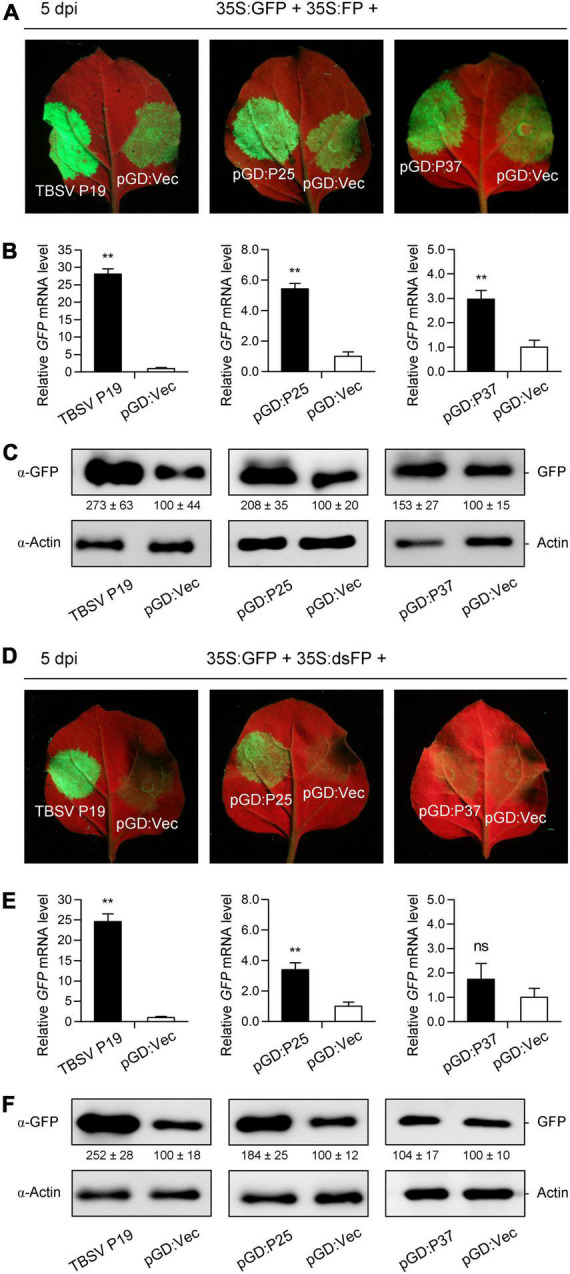
Firespike leafroll-associated virus (FLRaV) P25 and P37 can inhibit the post-transcriptional gene silencing (PTGS) of green fluorescent protein (GFP) mainly in a sense RNA manner. **(A)** Wild-type *Nicotiana benthamiana* seedlings were co-infiltrated with *Agrobacterium tumefaciens* containing the binary vector expressing GFP (35S:GFP) and 35S:FP (a C-terminal 400-bp fragment of sense GFP mRNA) plus pGD:Vec, TBSV P19, pGD:P25, or pGD:P37 at 5 days post infiltration (dpi). Leaves of N. benthamiana seedlings co-infiltrated with A. tumefaciens containing the 35S:GFP and 35S:FP plus pGD:Vec or TBSV P19 were served as negative and positive controls, respectively. **(B)** Quantitative real time PCR (qRT-PCR) analysis of the accumulation of GFP mRNA in leaf patches shown in **(A)**. *NbACT2* was utilized as the internal control. **(C)** Western blot (WB) analysis of the accumulation of GFP protein in leaf patches shown in **(A)**. GFP protein was detected using an antibody against GFP (α-GFP). **(D)** Wild-type *N. benthamiana* seedlings were co-infiltrated with *A. tumefaciens* containing the 35S:GFP and 35S:dsFP (an inverted repeat of C-terminal 400-bp GFP fragment) plus pGD:Vec, TBSV P19, pGD:P25, or pGD:P37 at 5 dpi. Leaves of *N. benthamiana* seedlings co-infiltrated with *A. tumefaciens* containing the 35S:GFP and 35S:dsFP plus pGD:Vec or TBSV P19 were served as negative and positive controls, respectively. **(E)** qRT-PCR analysis of the accumulation of *GFP* mRNA in leaf patches shown in **(D)**. *NbACT2* was utilized as the internal control. **(F)** WB analysis of the accumulation of GFP protein in leaf patches shown in **(D)**. GFP protein was detected using an α-GFP. For (B and E) Data are presented as means ± SD of three biological replicates. Means with asterisks are significantly different (Student’s *t*-test; *P* < 0.05). ns, not significant. For (c and f), WB analysis of actin with an α-Actin was served as the loading control.

Given that RNA silencing can be elicited by S-PTGS or IR-PTGS in plants (Li et al., 2014; Feng et al., 2018; Murai et al., 2022), we next investigated which form of PTGS could be inhibited by FLRaV P25 and P37. For this purpose, classic three-component S-PTGS and IR-PTGS assays were carried out as described previously (Li et al., 2014, 2015; Feng et al., 2018). In the S-PTGS assay, *N. benthamiana* leaves co-agroinfiltrated with 35S:GFP and 35S:FP plus pGD:Vec or TBSV P19 were served as the negative and positive controls, respectively. Compared with the negative control, *N. benthamiana* leaves co-agroinfiltrated with 35S:GFP and 35S:FP (a C-terminal 400-bp fragment of sense GFP mRNA) plus pGD:P25 or pGD:P37 showed strong GFP fluorescence around the infiltration sites, which were similar to that resulted from TBSV P19 (Figure 5A). To further confirm the observations detected in Figure 5A, qRT-PCR and WB analyses were conducted to determine the accumulation patterns of GFP at mRNA and protein levels. Consistently, the visible GFP fluorescence at the sites of infiltration of FLRaV P25 or P37 was because of the accumulation of GFP mRNA and protein (Figures 5B,C). These findings indicated that both FLRaV P25 and P37 were capable of inhibiting the sense *GFP* RNA-induced PTGS. In the IR-PTGS assay, *N. benthamiana* leaves co-agroinfiltrated with 35S:GFP and 35S:dsFP (an inverted repeat of C-terminal 400-bp GFP fragment) plus pGD:Vec or TBSV P19 were served as the negative and positive controls, respectively. As shown in Figure 5D, *N. benthamiana* leaves co-agroinfiltrated with 35S:GFP and 35S:dsFP plus pGD:P25 showed relatively strong GFP fluorescence around the infiltration sites. In contrast, *N. benthamiana* leaves co-agroinfiltrated with 35S:GFP and 35S:dsFP plus pGD:P37 exhibited undetectable GFP fluorescence around the infiltration sites, which was similar to that resulting from pGD:Vec (Figure 5D). As expected, compared with the positive control TBSV P19, there was moderate or no accumulation of GFP mRNA and protein at the sites of infiltration of FLRaV P25 or P37, respectively (Figures 5E,F). These results indicated that FLRaV P25 but not P37 was able to inhibit the IR-PTGS. Taken together, these results suggest that FLRaV P25 can suppress both the S-PTGS and the IR-PTGS and that FLRaV P37 is only able to inhibit the S-PTGS but not the IR-PTGS.

### FLRaV P25 functions as a suppressor of TGS of *GFP*

**FIGURE 6:**
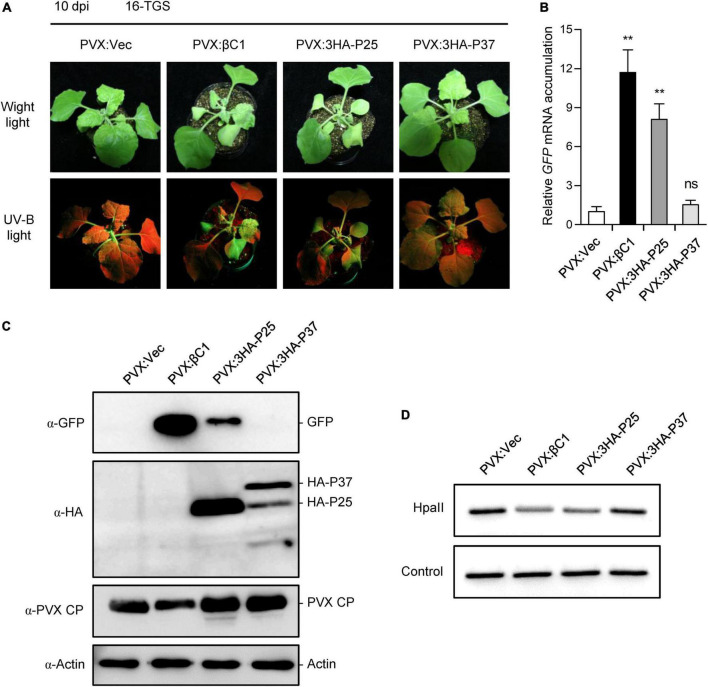
Firespike leafroll-associated virus (FLRaV) P25 and P37 can inhibit the transcriptional gene silencing (TGS) of green fluorescent protein (GFP) in 16-TGS *Nicotiana benthamiana*. **(A)** 16-TGS *N. benthamiana* seedlings were agroinfiltrated with an empty PVX vector (PVX:Vec), PVX:βC1, PVX:3HA-P25, or PVX:3HA-P37, and the seedlings at 10 days post infiltration (dpi). 16-TGS seedlings agroinfiltrated with PVX:Vec and PVX:βC1 were served as the negative and positive controls, respectively. **(B)** Quantitative real time PCR (qRT-PCR) analysis of the accumulation of *GFP* mRNA in systemic leaves shown in **(A)**. *NbACT2* was used as the internal control. Data are presented as means ± SD of three biological replicates. Means with asterisks are significantly different (Student’s *t*-test; *P* < 0.05). ns, not significant. **(C)** Western blot (WB) analysis of the accumulation of GFP protein in systemic leaves shown in **(A)**. GFP, HA-P25, HA-P37, and PVX CP were detected using antibodies to GFP (α-GFP), HA tag (α-HA), and to PVX-CP (α-PVX-CP), respectively. WB analysis of actin using an α-Actin was served as the loading control. **(D)** Chop-PCR analysis of the DNA methylation of the 35S promoter in 16-TGS. Genomic DNA was isolated from systemic leaves shown in **(A)**. Samples extracted from 16-TGS seedlings agroinfiltrated with PVX:Vec was served as the negative control.

To fully explore the biological functions of FLRaV P25 and P37, we next determine whether they can reverse the methylation-mediated TGS of GFP using 16-TGS plants (Buchmann et al., 2009; Zhong et al., 2017a). To this end, the ORFs of P25 and P37 genes of FLRaV were fused with a 3 × HA tag and cloned into a PVX-based expression vector to produce PVX:3HA-P25 and PVX:3HA-P37 constructs, respectively. The TGS assay was carried out as described previously (Zhong et al., 2017a; Zhao et al., 2022a). 16-TGS seedlings infiltrated with *A. tumefaciens* containing the empty PVX vector (PVX:Vec) or the TYLCCNB βC1 (PVX:βC1) (Zhong et al., 2017a) were served as the negative and positive controls, respectively. As anticipated, compared with the control, 16-TGS seedlings infiltrated with *A. tumefaciens* containing PVX:βC1 displayed extremely strong GFP fluorescence due to the reverse of the TGS-silenced GFP gene (Figure 6A). Similar to the PVX:βC1 positive control, relatively strong GFP fluorescence was also detected in 16-TGS seedlings infiltrated with *A. tumefaciens* containing PVX:3HA-P25 (Figure 6A), indicating that methylation-mediated TGS of *GFP* was reversed by FLRaV P25. In contrast, very feeble GFP fluorescence was detected in 16-TGS seedlings infiltrated with *A. tumefaciens* containing PVX:3HA-P37 (Figure 6A). To further confirm the GFP fluorescence observed in Figure 6A, qPCR and WB analyses were carried out to determine GFP accumulation patterns at the mRNA and protein levels. Consistently, the visible GFP fluorescence in 16-TGS seedlings infiltrated with FLRaV P25 or TYLCCNB βC1 was because of the accumulation of GFP mRNA and protein (Figures 6B,C). These results indicate that only FLRaV P25 is capable of reversing the established TGS-mediated silencing of *GFP* in 16-TGS plants.

Next, a general chop-PCR (Zhong et al., 2017a; Kong et al., 2020; Li et al., 2020) was performed to examine the 35S promoter methylation status in 16-TGS seedlings infiltrated with PVX:3HA-P25, PVX:3HA-P37 or the negative and positive controls (PVX:Vec and PVX:βC1), respectively. To this end, total genomic DNA was extracted from the systemic leaves of seedlings displayed in Figure 6A, and then digested with a methylation-sensitive endonuclease *Hpa*II and subjected to PCR. As shown in Figure 6D, similar to the positive control (PVX:βC1), amounts of the PCR products resulting from the 35S promoter in 16-TGS seedlings infiltrated with PVX:3HA-P25 were much lower than those derived from seedlings infiltrated with PVX:3HA-P37 or the negative control (PVX:Vec). This result indicated that the 35S promoter was demethylated by FLRaV P25 in 16-TGS seedlings infiltrated with PVX:3HA-P25. Collectively, these results suggest that FLRaV P25 can reverse established methylation-mediated TGS of *GFP* and act as a suppressor of TGS in *N. benthamiana*.

### FLRaV P25 aggravates the disease symptom and viral titer of PVX in *N. benthamiana*

**FIGURE 7:**
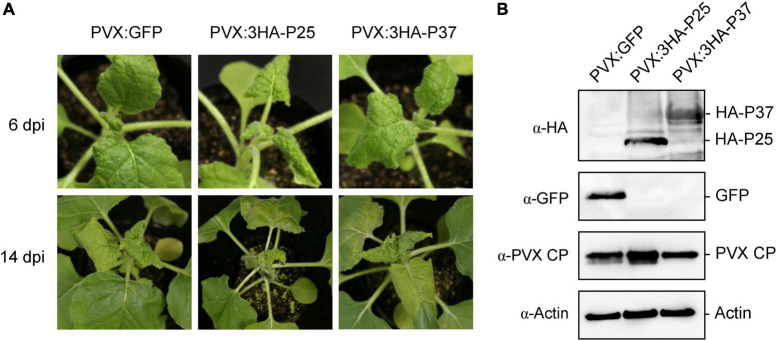
Effects of Firespike leafroll-associated virus (FLRaV) P25 and P37 on modulation of the symptom development and viral titer of potato virus X (PVX) in wild-type *Nicotiana benthamiana*. **(A)** *N. benthamiana* seedlings were agroinfiltrated with PVX:GFP, PVX:3HA-P25, or PVX:3HA-P37 at 6 days post infiltration (dpi) and 14 dpi. *N. benthamiana* seedlings agroinfiltrated with PVX: green fluorescent protein (GFP) were served as a negative control. **(B)** Western blot (WB) analysis of FLRaV P25 and P37 protein accumulation in systemic leaves shown in **(A)**. HA-P25, HA-P37, GFP, and PVX-CP were detected using antibodies to HA tag (α-HA), GFP (α-GFP), and to PVX CP (α-PVX CP), respectively. WB analysis performed using an antibody to actin was served as the loading control.

During the TGS assay, we noticed that 16-TGS seedlings agroinfiltrated with PVX:3HA-P25 or PVX:3HA-P37 showed leaf curl compared with the plants agroinfiltrated with PVX:Vec (Figure 6A), suggesting that FLRaV P25 and P37 may have a role in viral pathogenicity. To further evaluate these observations, we transiently expressed FLRaV P25 and P37 in wild-type *N. benthamiana* seedlings using the PVX-based expression system, and the PVX system expressing a GFP gene (PVX:GFP) was served as a negative control. As expected, compared with *N. benthamiana* seedlings agroinfiltrated with PVX:GFP, seedlings agroinfiltrated with either PVX:3HA-P25 or PVX:3HA-P37 displayed severe leaf curl at 6 dpi (Figure 7A, top panel). Interestingly, at 14 dpi, *N. benthamiana* seedlings agroinfiltrated with PVX:3HA-P25 exhibited unexpected premature leaf deaths and moderate leaf curl, whereas *N. benthamiana* seedlings agroinfiltrated with PVX:3HA-P37 displayed very slight leaf curl that was similar to these agroinfiltrated with PVX:GFP (Figure 7A, bottom panel). WB analysis with an anti-HA verified the expression of FLRaV P25 and P37 in the upper non-infiltrated *N. benthamiana* leaves at 14 dpi (Figure 7B, top panel), suggesting that FLRaV P25 and P37 were accurately maintained in viral progenies. Furthermore, WB analysis with an anti-PVX CP antibody showed that PVX accumulation in the upper leaves of PVX:3HA-P25-infected *N. benthamiana* seedlings was higher than that in the *N. benthamiana* leaves agroinfiltrated with PVX:3HA-P37 or PVX:GFP (Figure 7B, middle panel), indicating that FLRaV P25 may help induce the accumulation and viral titer of PVX. Collectively, these results suggest that FLRaV P25 but not P37 aggravates the disease symptom and viral titer of PVX in *N. benthamiana*.

## Discussion

We have previously identified and characterized a novel FLRaV in firespike and found that it is capable of infecting *N. benthamiana* through mechanical inoculation and infectious cDNA clones (Wang et al., 2020). Here, we reported the characterization of FLRaV P25 and P37 as VSRs that were actively involved in suppressing both the PTGS and TGS.

In plants, it has been shown that RNA silencing is a crucial immune system to preventing viral infections (Yang and Li, 2018; Li et al., 2019; Jin et al., 2021, 2022). To overcome the host immune system, plant viruses have developed various VSRs to inhibit RNA silencing at different steps (Feng et al., 2018; Guo et al., 2019; Jin et al., 2021, 2022). Previous studies have shown that the most common counteraction of VSRs is to suppress vsiRNA amplification (Guo et al., 2019; Li and Wang, 2019; Jin et al., 2022). In the present study, we demonstrated that FLRaV P25 could suppress PTGS at both the S-PTGS and the IR-PTGS levels (Figures 3, 5). In contrast, FLRaV P37 could only inhibit the S-PTGS but not the IR-PTGS (Figure 5). These findings agree with previous reports which showed that VSRs can target not only the production of dsRNA formation but also the actions of secondary siRNAs in the RNA silencing pathway (Silhavy et al., 2002; Bayne et al., 2005; Li et al., 2015; Feng et al., 2018; Zhao et al., 2022b).

As well known, RNA silencing in plants can be divided into three main stages, that is silencing initiation, the spread of the silencing signal, and maintenance of silencing (Feng et al., 2018; Zhao and Guo, 2022). Recently, several plant virus-encoded VSRs, which include cucumber mosaic virus (CMV) 2b, rice stripe virus (RSV) NS3, and strawberry vein banding virus (SVBV) P6, have been found to be involved in suppressing systemic RNA silencing in plants (Ghosh et al., 2021). CMV 2b is one of the first VSRs that are demonstrated to prevent the RNA silencing in systemic leaves, probably by impeding the initiation of systemic propagation of the silencing signal (Xu et al., 2013; Du et al., 2014). RSV NS3 can suppress systemic RNA silencing by sequestering siRNA duplexes, interfering with dsRNA production, and further blocking the spread of the silencing signal (Xiong et al., 2009; Zheng et al., 2017). As a VSR, SVBV P6 can suppress systemic RNA silencing by impeding the spread of the silencing signal (Feng et al., 2018; Li et al., 2018b). Here, we demonstrated that although FLRaV P25 and P37 could not prevent the short-range movement of the silencing signal, they were capable of preventing the RNA silencing signal from spreading systemically in 16c *N. benthamiana* (Figure 4). This finding corroborates previous reports which showed that VSRs from the genus *Ampelovirus* of *Closteroviridae* family are frequently capable of inhibiting the systemic RNA silencing in 16c *N. benthamiana* (Kubota and Ng, 2016; Li et al., 2018a; Zhang et al., 2020).

Over the past two decades, it has been well demonstrated that host RNA-directed DNA methylation (RdDM)-mediated TGS is one of the most effective defense responses against geminiviruses due to their DNA replication in the host nucleus (Raja et al., 2008; Zhong et al., 2017b; Zarreen and Chakraborty, 2020; Gupta et al., 2021). In turn, geminiviruses evolve various VSRs to interfere with the host RdDM-mediated TGS (Hanley-Bowdoin et al., 2013; Wang et al., 2019; Jin et al., 2021; Zhou, 2021). Interestingly, several RNA viruses encoded VSRs are found to be able to inhibit RdDM-mediated TGS in host plants (Zhao et al., 2016; Jin et al., 2021). For example, the CMV 2b, which is originally identified as a viral suppressor of PTGS, primarily localizes in the nucleolus of the host cell and exhibits a siRNA-binding ability that is sufficient to inhibit the RdDM-mediated TGS (Duan et al., 2012). Consistently, CMV 2b-transgenic and CMV-infected *Arabidopsis* plants show reduced DNA methylation levels at a genome-wide level (Zhao et al., 2018). Similarly, VSR HC-Pro from tobacco vein banding mosaic virus (TVBMV) has been demonstrated to be able to reduce the methylation in the promoter region of a set of host genes implicated in salicylic acid and auxin biosynthesis pathways (Yang et al., 2016, 2020). In this study, we demonstrated that FLRaV P25 was capable of reversing the established methylation-mediated TGS of GFP in 16-TGS *N. benthamiana* (Figure 6). We also showed that FLRaV P25 was localized in both cytosol and nucleus (Figure 1), which provides a spatial possibility for its inhibition function on TGS. This finding corroborates previous reports which demonstrated that RNA virus-encoded VSRs can bind siRNAs and suppress RdDM-mediated TGS in the nucleus (Nuthikattu et al., 2013; Zhao et al., 2016).

In addition to suppressing RNA silencing, many VSRs are also characterized as determinants of viral symptoms in host plants (Feng et al., 2018; Hussain et al., 2021). Transient and transgenic expression of RSV NS3, CMV 2b, SVBV P6, or TVBMV HC-Pro in *Arabidopsis* or *N. benthamiana* plants result in severe chlorosis, mosaics, or leaf curl on leaves (Xiong et al., 2009; Yang et al., 2016; Feng et al., 2018; Zhao et al., 2018). In our study, when FLRaV P25 was transiently expressed in *N. benthamiana* using a PVX-based expression system, much severe leaf curl and premature leaf deaths were detected in the PVX:3HA-P25-infiltrated plants (Figure 7). This might because that the accumulation of FLRaV P25, we speculated that the enhanced symptoms displayed in PVX:3HA-P25-infiltrated *N. benthamiana* resulted from increased PVX replication in cells following the suppression of RNA silencing by FLRaV P25. Therefore, future studies should focus on how FLRaV P25 aggravates the disease symptom of PVX so the mechanisms underlining RNA virus-encoded VSR functions in viral pathogenicity of the genus *Ampelovirus* of *Closteroviridae* family.

In conclusion, our results demonstrate that FLRaV P25 and P27 proteins have functional suppression activities on host PTGS and TGS, which may interfere with host RNA silencing during viral infection. To the best of our knowledge, this is the first report which characterizes the functions of FLRaV P25 and P27 proteins as VSRs. Furthermore, further studies should focus on how FLRaV P25 aggravates the disease symptom of PVX in the future.

## Data availability statement

The original contributions presented in the study are included in the article/Supplementary material, further inquiries can be directed to the corresponding authors.

## Author contributions

TH and XZ conceived the study. YW, HL, and YG conducted experiments. YW and TH analyzed experimental data. YW, ZW, and XZ wrote the manuscript. All authors read and agreed, and gave final approval to the published version of the manuscript.
